# Neurophysiology of Grasping Actions: Evidence from ERPs

**DOI:** 10.3389/fpsyg.2016.01996

**Published:** 2016-12-22

**Authors:** Dirk Koester, Thomas Schack, Jan Westerholz

**Affiliations:** ^1^Center of Excellence – Cognitive Interaction Technology (CITEC)Bielefeld, Germany; ^2^Neurocognition and Action – Biomechanics Research Group, Faculty of Psychology and Sport Science, Bielefeld UniversityBielefeld, Germany; ^3^Research Institute for Cognition and Robotics (CoR-Lab)Bielefeld, Germany

**Keywords:** EEG, event-related potentials, manual action, cognition

## Abstract

We use our hands very frequently to interact with our environment. Neuropsychology together with lesion models and intracranial recordings and imaging work yielded important insights into the functional neuroanatomical correlates of grasping, one important function of our hands, pointing toward a functional parietofrontal brain network. Event-related potentials (ERPs) register directly electrical brain activity and are endowed with high temporal resolution but have long been assumed to be susceptible to movement artifacts. Recent work has shown that reliable ERPs can be obtained during movement execution. Here, we review the available ERP work on (uni) manual grasping actions. We discuss various ERP components and how they may be related to functional components of grasping according to traditional distinctions of manual actions such as planning and control phases. The ERP results are largely in line with the assumption of a parietofrontal network. But other questions remain, in particular regarding the temporal succession of frontal and parietal ERP effects. With the low number of ERP studies on grasping, not all ERP effects appear to be coherent with one another. Understanding the control of our hands may help to develop further neurocognitive theories of grasping and to make progress in prosthetics, rehabilitation or development of technical systems for support of human actions.

## Introduction

Our hands are important tools to interact with our environment. Yet, we do not fully understand the biological underpinnings of manual actions, that is, how the central nervous system (CNS) achieves and controls manual dexterity. In motor control, it remains a debated issue whether or not the brain entertains (stored) grasp representations (i.e., grip types). Otherwise, grasping movements might be computed on the fly when needed. Here, we take an empirical perspective on neurophysiological processes underlying grasping actions.

How we grasp an object is determined by external and by internal factors. External factors refer to object properties, such as size, weight, shape, or texture (intrinsic properties) and situation aspects, such as position, distance (i.e., spatial coordinates) or possibly orientation of the object (extrinsic object properties; cf. dual-channel hypothesis; Jeannerod, 1981; Castiello, 2005; Goodale, 2011). In contrast, internal factors are properties of the person or the task; that is, largely cognitive variables, such as action planning, the goal of the action, habits or familiarity with the object or action (Rosenbaum et al., 1992; Meulenbroek et al., 1993; Kunde et al., 2007; van der Wel et al., 2007; Logan, 2009; Herbort and Butz, 2010, 2011; Knudsen et al., 2012; Belardinelli et al., 2016). For example, when grasping a bottle, the hand movement including the shaping of the fingers differs depending on whether the bottle will be thrown or precisely placed (Ansuini et al., 2008). That is, the purpose or goal of the action influences the (preceding) movement (Marteniuk et al., 1987; Armbrüster and Spijkers, 2006; Rizzolatti et al., 2014) which reflects a cognitive influence on grasping movements (see also Glover, 2004; Seegelke et al., 2014). Cognitive processes are of particular interest; they are subserved by cortical activity. Electroencephalography (EEG) and magnetoencephalography (MEG) are very well-suited to examine cortical activity in normal healthy humans, especially when fast (cognitive) processes are of interest.

For the performance of such actions, two processing stages have been distinguished (two-component model; Woodworth, 1899; Elliott et al., 2001). In a planning phase, the movement is prepared and initiated without sensory feedback. In the control phase, the movement execution is monitored including (re-)afferent feedback information (Wolpert and Ghahramani, 2000; Glover et al., 2012). An overarching question is whether or not such grip types are (neuro) cognitively represented (Rizzolatti et al., 2000; Begliomini et al., 2007). Alternatively, grasping movements may be computed (e.g., as contact points for the fingers; Smeets and Brenner, 1999), or movement profiles might result from tissue and mechanical properties of the movement system (e.g., Kelso, 1994, 1997). Here, we are concerned with the neurophysiological correlates of (uni) manual grasping in humans with a focus on event-related potentials (ERPs) as there is little such research available.

Other neurocognitive methodologies have been applied in manual action research of which grasping is one important type and these have their own limitations (see below). So far, there is agreement regarding a parietofrontal brain network as the neuroanatomical correlates of grasping (Castiello, 2005; Filimon, 2010; Grafton, 2010 for review), but many neurophysiological studies focussed on reaching or tool use rather than grasping (e.g., Wheaton et al., 2005a; Archambault et al., 2009; Proverbio et al., 2011; Striemer et al., 2011). Other manual actions also received some attention in neurophysiological research, for example, finger or arm movements (Wessel et al., 1994; Slobounov et al., 1998), tapping (Lang et al., 1990), and throwing (Frömer et al., 2012).

Different parts of the parietofrontal network are assumed to fulfill different functions in manual actions. For reaching, it is assumed that parietal areas, especially the parieto-occipital sulcus represent the goals of the movement (Astafiev et al., 2003; Hamilton and Grafton, 2006). Other authors assume that visuo-spatial information regarding the target objects are processed in parietal areas (Jeannerod et al., 1995; Galletti et al., 2003; Filimon et al., 2009; Tarantino et al., 2014) whereas Goldenberg and Spatt (2009) attribute the processing of mechanical interactions among effectors and the environment to parietal areas.

More consistently, frontal areas are proposed to subserve executive functions, and some specifically ascribe goal representations (van Elk et al., 2012) to frontal areas, the planning of sequential behavior (van Schie and Bekkering, 2007) or the control of action execution (Haggard, 2011; Ridderinkhof et al., 2004). Furthermore, in primate studies which combined reaching and grasping, so-called motor schemas have been assigned to frontal areas whereas perceptual schemas have been ascribed to parietal areas (Rizzolatti et al., 1988; Jeannerod et al., 1995). More recently, two distinct neural processing streams have been discussed; one for the transport and one for the grasping component (Jeannerod, 1984, 1988; Galletti et al., 2003; Hattori et al., 2009; Vesia et al., 2013). However, Fattori et al. (2010) reported grasping (pre-shaping of the hand) specific neurons in the “transport stream” of macaque monkeys, specifically, in the medial posterior aspect of the parietal area (V6A, see also Galletti et al., 1999). That is, while there is some agreement about the functional role of frontal areas for manual actions, there is more of a controversy regarding parietal brain areas.

Because movements themselves can pose recording problems for EEG and MEG, such studies often used simple movements like button presses or have focused on the planning/preparation phase of a movement or imagined movements (Verleger et al., 2000; Zaepffel and Brochier, 2012). However, recently reliable ERPs with their high temporal resolution (msec range) have been obtained during movement execution (van Schie and Bekkering, 2007; Koester and Schiller, 2008; Kirsch and Hennighausen, 2010) and are, thus, suitable for investigating the neurophysiological underpinnings of manual actions; it appears that a sufficient number of artifact free trials could be obtained in these studies (for approaches to correct for movement artifact see, e.g., Hesse and James, 2006; Ting et al., 2006). So far, only very few ERP studies have investigated grasping movements. Examining the temporal dynamics of the underlying neurophysiology can advance our understanding of the biological underpinnings of motor control and their links to cognition.

The available grasping ERP studies investigated the neural correlates of different grip types (precision and power grips), for example, with an emphasis on immediate or final action goals. Others studied the relation between neurophysiological processes and behavioral consequences and co-registered kinematic and ERP data. Also, the relation between symbolic processing and grasping has been addressed by using language materials as imperative cues for grasping responses. Also, the role of intentions and habitual effects were investigated in grasping.

van Schie and Bekkering (2007) reported, as far as we know, for the first time ERP findings on executing precision grips. They had participants grasp and transport an object across or below a barrier instructing either how to grasp the object (immediate goal) or where to place the object (final goal). Both, immediate and final goal instructions required the same movements excluding explanations by kinematic differences. Hence, the ERP effects, namely a parieto-occipital slow wave related to the immediate goal and a slow wave over left frontal regions for the final goal specification have been related to the prehension of the object and the planning/control of sequential behavior, respectively. Consistent with the posterior effects, van Elk et al. (2012) reported enhanced parietal activation for the *observation* of grip errors and interpreted them as processing hand-object interaction. Westerholz et al. (2013) extended van Schie and Bekkering’s (2007) study to power grips (also comparing the same movements emphasizing the immediate or the final goal) to test for differences in the neurophysiological time course because power grips are known to have a shorter deceleration phase and a later maximum grip aperture than precision grips (Castiello et al., 1992).

Both precision (van Schie and Bekkering, 2007) and power grips (Westerholz et al., 2013) yielded a temporally similar frontal slow wave (-1,100 to 0 ms; bilateral for power grips) time-locked to movement end. If reflecting the planning/control of sequential behavior, these planning/control processes should not differ for the grip types. The planning might relate to the goal state (or posture) because the ERP effects were time-locked to the movement end. In contrast, precision and power grips differed temporally over posterior areas. Power grips elicited a posterior slow wave (from at least -600 to -200 ms time-locked to grasping) that *preceded* the posterior slow wave for precision grips by about 300 ms (-300 to 0 ms). This posterior effect was related to the prehension, and as it precedes the grasping itself, it may, specifically, reflect the preparation of the prehension. Such a preparation may be easier for power compared with precision grips as fewer parameters have to be controlled with less precision.

Another study combined kinematic (hand position and grip aperture) and ERP measures in grasping (De Sanctis et al., 2013). Participants grasped small or large objects with a precision or a power grip, respectively, while kinematics and ERPs were recorded. An increased negativity (motor-related N400) was found at frontal midline electrodes for grasping small objects compared with larger objects. Importantly, this m-N400 component peaked earlier for power than precision grips in accordance with the time course differences between these grip types reported above (van Schie and Bekkering, 2007; Westerholz et al., 2013). De Sanctis et al. (2013) reported ERP amplitude effects before (planning) and during movement execution. Furthermore, movement time (kinematics) and m-N400 latency (ERPs) were positively correlated for both grip types (*r* = 0.46 and 0.49) suggesting a functional relation between neural and behavioral components of grasping actions.

Another study that co-registered kinematics and ERPs investigated whether grasping is influenced by word reading (Boulenger et al., 2008). Participants were asked to perform a reach-and-grasp task in response to written (non) words, and for the critical results the ERPs and the kinematics for verbs and nouns were compared. (Verbs but not nouns were expected to pre-activate neural circuits for motor control because only verbs refer to actions.) Even though Boulenger et al. (2008) did not investigate various grip types, the results are partly in line with De Sanctis et al.’s (2013) data in that the ERPs showed a reduced ERP amplitude (taken to be the *readiness potential*, a motor planning ERP component; Kornhuber and Deecke, 1965) for verbs compared with nouns before movement initiation, that is, during planning; the ERP was also affected during movement execution. That is, the ERPs were affected in both phases, planning and execution, as in De Sanctis et al.’s study. Boulenger et al. (2008) reported also a kinematic effect; the wrist’s maximal acceleration was smaller for the verb compared to the noun condition but the ERPs and the kinematics were not analyzed in a combined manner.

Shared neural mechanisms of word reading and grasp planning (in delayed action execution) were also investigated by van Elk et al. (2008). In a dual-task paradigm, participants prepared grasping one of two objects (magnifying glass or cup). Then a semantic categorisation was performed on a written word (to ensure deep processing; Collins and Loftus, 1975). After a subsequent and delayed go/no-go stimulus (a tone), the prepared action was performed in go trials. Critically, two factors were manipulated. First, the action could be meaningful (bringing the glass toward the eye or the cup toward the mouth) or meaningless (glass to mouth or cup to eye). Second, the words could be congruent (“eye” or “mouth”) or incongruent (e.g., “belly” or “knee”) with the action goal. (All critical words referred to body parts.) Similar to Boulenger et al. (2008), the results suggest that reading and action planning share some neural resources. In particular, words that were incongruent with the action goal elicited an anterior N400 effect compared with congruent words but only if the prepared action was meaningful. If the prepared action was meaningless, no N400 effect was obtained. Even though it remains to be shown whether these effects can be generalized to the execution phase, the results go beyond Boulenger et al.’s findings, as they show an involvement of semantic information in preparing a grasping action whereas Boulenger et al., reported an effect for word class, that is, syntactic information.

Interestingly, the N400 effects had a more frontal focus than is reported in language studies (Kutas and Federmeier, 2011). This distribution is in accordance with the frontal distribution in the study by De Sanctis et al. (2013) and has also been found in *spoken* language production (Koester and Schiller, 2008). These initial observations raise the possibility that such an anterior distribution is related to frontal, maybe (pre)motor, and cortical generators.

Clearly, influences of symbolic information (e.g., reading words) on grasping are cognitive in nature. Three other studies investigated the influence of intentions on grasping by means of ERPs. Two studies investigated the preparation phase of precision grips, and one the planning and execution of power grips. To investigate temporal organization of action planning, Bozzacchi et al. (2012a) recorded pre-movement ERPs, specifically the readiness potential (RP). Participants had three different tasks; reaching, grasping and impossible grasping (attempting to grasp with constrained fingers) of a teacup. These authors found an earlier RP onset over parietal areas and a later RP onset over frontal areas. Parietal effects have been suggested to reflect the specification of grip-related information (i.e., preparation of prehension; van Schie and Bekkering, 2007) that is further processed in frontal areas (Bozzacchi et al., 2012a; see Wheaton et al., 2005a,b, 2009 for manual pantomime movements). The activity over parietal areas began well before action onset for grasping, but not for reaching or impossible grasping. More interestingly, premotor activity seems to precede parietal activity in some instances (Filimon, 2010), but Bozzacchi et al. (2012a) reported parietal activity to precede frontal activity which could be related to the temporal order of parietal before frontal effects in van Schie and Bekkering (2007) and Westerholz et al. (2013; see also Wheaton et al., 2005a). Thus, the temporal organization of the neural mechanisms underlying grasping and its preparation remains controversial.

Similarly, Bozzacchi et al. (2012b) compared ERPs for real grasps, “virtual” grasps (key presses that released a video showing a grasping action) and key presses (without a subsequent effect). Similar motor preparation processes (RP) were found for real and virtual grasps over posterior parietal areas which differed from the key press condition. Again, the parietal activity for virtual and real grasps preceded activity over (pre)motor areas. As the ERP for virtual grasping resembled the real grasping and not the key press condition, Bozzacchi et al. (2012b) conclude that the goal of the action and not the movement kinematics influenced the preparation phase. Furthermore, these authors suggested that action preparation is affected by the meaning of that action, similar to the overall conclusion by van Elk et al. (2008). That is, Bozzachi et al.’s works point toward an influence of the anticipated goal state on grasping preparation. Zaepffel and Brochier (2012) used the contingent negative variation (CNV) to investigate planning processes for a grasping task (reach-grasp-and-pull). Although these authors did not report ERP effects for execution, their findings are taken to support goal anticipations (force and hand shape, i.e., grip type) in accordance with Bozzacchi et al.’s works.

Similar to anticipations, Westerholz et al. (2014a) investigated the role of intentional processes in grasping, that is, the decision what action to perform. These authors investigated the neurophysiological correlates of planning and execution of goal-related grasping and the dominance of an immediate “goal” (grip configuration) and a final goal (goal state). They used a bar transport task; participants grasped a horizontally suspended bar and placed it on one of two target positions. Depending on the experimental condition either the grip (underhand vs. overhand grip) or the goal state (target position and bar orientation) were specified. Alternatively, grip or goal state could be free (i.e., unspecified) and participants had to decide for themselves (2 × 2 design). Interestingly, the specification (i.e., instruction) of goal states yielded a parietal slow wave in the ERP that was extended toward frontocentral electrodes time-locked to grasping. Also, a right frontal slow wave was found time-locked to movement end. These effects were tied to (prepared) prehension and the planning or anticipation of an action sequence, respectively (see van Schie and Bekkering, 2007; Westerholz et al., 2013). Critically, these ERP effects were elicited by the specification of the goal. The specification of the grip itself did *not* yield any ERP effect suggesting that the goal state which needs to be anticipated is more important than the immediately required movement for grasping, underlining the importance of anticipation in grasping in line with Bozzacchi et al. (2012a,b) and Zaepffel and Brochier (2012).

In another task (grasp-and-rotate), Westerholz et al. (2014b) investigated the role of habitual action control in addition to specified vs. free grip choices. Habitual influences on action have been suggested next to goal state anticipations by Herbort and Butz (2011; see also Künzell et al., 2013). Westerholz et al. (2014b) asked participants to grasp a bar that was marked at one end (as a pointer) and mounted to a dial. When grasping, participants had to rotate the dial immediately. The concomitantly recorded ERPs were analyzed for specified vs. free grips and also for habitual vs. non-habitual grasping (thumb toward the pointer end is considered habitual; Rosenbaum et al., 1992). Interestingly, a bilateral frontal slow wave time-locked to movement end was more negative for non-habitual grasping but there were no posterior ERP effects. Similar to Westerholz et al. (2014a), grip specification yielded also no ERP effects. Whether the absence of posterior effects is related to a “missing” transport phase, to the nature of the task (rotation vs. transport) or the functionality of the rotation (Tucker and Ellis, 1998) remains to be shown. For an overview of these ERP studies on grasping see **Table 1**.

**Table 1:** Overview of event-related potential (ERP) studies on grasping (grip execution) including tasks and major outcomes.

	Task	Grip type	Condition	Effect	Distribution
van Schie and Bekkering, 2007	Grasp and transport	Precision	Final goal vs. immediate goal emphasis	Negativity (for final goal emp.)	Left frontal
				Positivity (for final goal emp.)	Parieto-occipital
Westerholz et al., 2013	Grasp and transport	Power	Final goal vs. immediate goal emphasis	Negativity (for final goal emp.)	Right frontal
				Positivity (for final goal emp.)	Parieto-occipital
De Sanctis et al., 2013	Reach-to-grasp	Precision/power	Small vs. larger object	N400 latency and amplitude (precision later and more negative)	Frontal
				P300 amplitude (power > precision)	Parietal
Boulenger et al., 2008	Go/No-go (LDT and Reach and grasp)	Pinch	Word category/action match (verb, noun vs. non word)	RP gradient (reduced for verbs vs. nouns)	Central
van Elk et al., 2008	Go/No-go (semantic categorization and grasping)	Object-specific (magnifying glass/cup)	Noun/object (i.e., grip) match	N400 amplitude (incongruent > congruent for meaningful actions)	Frontal focus
Bozzacchi et al., 2012a	(Impossible) grasping	Object-specific (cup)	Constraint fingers (vs. free)	(posterior) RP^∗^ (grasping more negative than impossible)	Parietal
				(anterior) RP^∗^ (earlier and and more negative grasping vs. impossible)	Frontal-central
Bozzacchi et al., 2012b	(Virtual) grasping	Object-specific (cup)	Grasping video vs. grasping	RP^∗^ (similar for virtual and actual grasping vs. key press)	Posterior parietal [before (pre) motor]
Westerholz et al., 2014a	Grasp and transport	Power	Goal specification (vs. free)	Negativity (for free goals)	Right frontal
				Positivity (for free goals)	Central
Westerholz et al., 2014b	Grasp and rotate	Power	Specified (vs. habitual)	Negativity	Frontal
Zaepffel and Brochier, 2012 (only planning)	Reach-grasp-pull	Precision/side grip	Increased amount of information (precuing)	Topographical variation of the late CNV	

*LDT, lexical decision task; RP, readiness potential; CNV, contingent negative variation. ^∗^Bozzacchi et al., use the term Bereitschaftspotential for the RP.*

As said, other approaches have been applied to manual actions (including grasping). The EEG signal offers further approaches for experimental design and signal analysis not reviewed here. Analyses of various frequency bands of the EEG signal (e.g., Wheaton et al., 2009), of the lateralized readiness potentials (LRP; Verleger et al., 2000; Leuthold and Jentzsch, 2001) and the CNV are fruitful approaches (Zaepffel and Brochier, 2012). Further neurocognitive methodologies that have been used comprise lesion studies, intracranial recording (predominantly animals; e.g., Galletti et al., 2003; Fattori et al., 2010), neuropsychological studies (predominantly human patients; e.g., Wessel et al., 1994; Verleger et al., 1999, 2003; Goldenberg and Spatt, 2009) and functional brain imaging (blood flow changes; e.g., Burnod et al., 1999; Majdandzić et al., 2007; Piefke et al., 2009; Andric et al., 2013). As all others, these methodologies have also different limitations. Integration of these methodologies would provide valuable insights but has to await future research.

Future work may also investigate the role of other modalities (e.g., tactile or haptics). The relation between manual control and other cognitive domains (e.g., language or memory) is not fully understood (Pulvermüller, 2005; Spiegel et al., 2013; Zhang et al., 2014). Neurocognitive models of grasping would benefit from relating ERPs to other imaging methods, for example, by localization analyses to establish a functional link between functional anatomical correlates and ERP effects. Finally, future work may focus on applications, for example, in sports (e.g., Pezzulo et al., 2010; Bläsing et al., 2014), in the medical realm (e.g., rehabilitation or for prosthetics) or for improving technical support systems (i.e., human machine interactions; Schack and Ritter, 2013).

Taken together, this review suggests that ERPs can be used reliably to investigate the neurophysiological correlates of grasping movements. The reviewed ERP studies consistently report anterior and posterior (slow) ERP amplitude modulations in line with the evidence for a parietofrontal brain network obtained with other methodologies (Grafton, 2010). As the interpretations of the ERP effects diverge and the number of ERP studies is still relatively low, more research is needed to resolve apparent inconsistencies, for example, regarding the temporal succession of parietal and frontal processes. The combination and extension of methodologies promises to be a fruitful line of action to better apprehend prehension.

## Author Contributions

DK, TS, and JW conceptualized and wrote the manuscript.

## Conflict of Interest Statement

The authors declare that the research was conducted in the absence of any commercial or financial relationships that could be construed as a potential conflict of interest.
